# AI-Enhanced Thermal–Visual–Inertial Odometry and Autonomous Planning for GPS-Denied Search-and- Rescue Robotics

**DOI:** 10.3390/s26082462

**Published:** 2026-04-16

**Authors:** Islam T. Almalkawi, Sabya Shtaiwi, Alaa Alhowaide, Manel Guerrero Zapata

**Affiliations:** 1 Computer Engineering Department, Faculty of Engineering, The Hashemite University, Zarqa 13133, Jordan; sabyashtaiwi4@gmail.com; 2 Department of Computer Information Systems, Faculty of Computer and Information Technology, Jordan University of Science and Technology, Irbid 22110, Jordan; azalhowaide@just.edu.jo; 3 Computer Architecture Department, Universitat Politècnica de Catalunya, 08034 Barcelona, Spain; manel.guerrero-zapata@upc.edu

**Keywords:** search and rescue robots, GPS-denied environments, multi-sensor fusion, autonomous navigation, deep reinforcement learning

## Abstract

Search and rescue (SAR) missions in collapsed or underground environments remain challenging due to GPS unavailability, which hinders localization and autonomous navigation. Systems that rely on single-sensor inputs or structured settings often degrade under smoke, dust, or dynamic clutter. This paper presents an autonomous ground robot for GPS-denied SAR that integrates low-cost thermal, visual, inertial, and acoustic cues within a unified, computation-efficient architecture. The stack combines Thermal–Visual Odometry (TV–VO) with Zero-Velocity Updates (ZUPT) for drift-resistant localization, RescueGraph for multimodal survivor detection, and a Proximal Policy Optimization (PPO) planner for adaptive navigation under uncertainty. Across simulated disaster scenarios and benchmark corridor runs, the system shows embedded-feasible runtime behavior and supports return to base without external beacons under the evaluated conditions. Quantitatively, TV–VO+ZUPT reduces drift in short internal evaluations, while RescueGraph attains an F1-score of 0.6923 and an area under the ROC curve (AUC) of 0.976 for survivor detection. At the system level, the integrated navigation stack achieves full mission completion in the reported SAR-style trials, while the separate A*/PPO comparison highlights a trade-off between completion rate, traversal time, and collisions. Overall, the results support the practical promise of a low-cost sensor-fusion and learning-assisted navigation framework for GPS-denied SAR robotics.

## 1. Introduction

In disaster management, search and rescue (SAR) operations are extremely time critical, as even short delays can determine survival outcomes. In collapsed or smoke-filled zones, GPS-based navigation becomes unreliable or unavailable, motivating autonomous robots to assist first responders and enhance situational awareness [1,2].

Reliable localization in GPS-denied environments remains challenging. Most existing methods are based on single-sensor modalities such as monocular vision or depth estimation, which fail under low visibility or clutter [3]. Learning-based navigation has been explored [4], yet high computational costs limit embedded deployment.

Multimodal systems like TPL-SLAM [5] and LiDAR Stereo Thermal fusion [6] offer accurate tracking under smoke or fog but rely on expensive sensors and complex calibration. In contrast, this work targets low-cost, computation-efficient fusion optimized for embedded SAR robots.

Prior studies achieved either high accuracy in structured environments [3] or adaptive, learning-based planning [4], but integrated frameworks that jointly combine multi-sensor fusion and computation-aware adaptability for GPS-denied SAR remain limited [7]. Bridging this gap is essential to balance resilience, adaptability, and efficiency in real-world rescue missions.

This paper introduces an AI-powered ground SAR robot that integrates thermal, visual, and inertial sensing with deep reinforcement learning (DRL) for adaptive navigation. The framework prioritizes robustness and real-time efficiency on resource-constrained hardware.

The main contributions are as follows:

- A tightly coupled multi-sensor fusion framework for robust, drift-resistant localization in GPS-denied environments.
- An adaptive DRL-based navigation system resilient to uncertainty and dynamic obstacles.
- A computation-aware design targeting embedded-feasible operation on resource-constrained processors.
- Comprehensive validation in simulated disaster scenarios that approximate field conditions.

To clarify the scope of our contribution, this work does not claim a fundamentally new SLAM, Extended Kalman Filter (EKF), or graph search algorithm in isolation. Rather, its main contribution lies in the integration, adaptation, and evaluation of established sensing, fusion, detection, and planning components within a unified low-cost autonomy stack for GPS-denied SAR operation.

The remainder of this paper is organized as follows. Section 2 reviews recent advances in SAR robotics. Section 3 outlines methodology and system design. Section 4 details sensing, perception, mapping, and planning. Section 5 discusses implementation and prototype readiness. Section 6 describes datasets and evaluation protocols. Section 7 reports results, followed by ablation studies in Section 8. Section 9 provides analysis, and Section 10 concludes the paper.

## 2. Related Work

Autonomous search and rescue (SAR) robotics in GPS-denied environments as a topic has received growing attention across ground perception, UAV autonomy, and multi-robot coordination frameworks. Table 1 summarizes key approaches and their limitations relative to the proposed design.

Rado et al. [8] introduced an adaptive vision-based navigation method for cluttered post-disaster environments, achieving strong obstacle handling but with high computational cost. Henriques [9] employed camera-based mapping for ground and aerial robots using low-cost sensors, though performance degraded under smoke or low illumination. Jadeja et al. [10] applied deep learning for survivor detection on snake robots with high mobility in confined rubble, yet they were limited by mechanical stability and energy use. Schichler et al. [3] demonstrated robust thermal visual fusion for tunnel localization in GNSS-denied conditions but still required precise synchronization and calibration. Allan and Barczyk [1] developed a cost-effective quadcopter that was validated experimentally but constrained by endurance and payload, while Jarraya et al. [4] analyzed UAV sensor fusion trade-offs, noting real-time computational limits. Bravo–Arrabal et al. [7] proposed a 6G-enabled multi-robot SAR framework that offered scalability but was reliant on emerging communication infrastructure. Similarly, Tao et al. [11] demonstrated air–ground collaborative map fusion with PPO-based navigation, achieving strong coverage but requiring aerial platforms that are unavailable in lightweight ground deployments.

Beyond SAR-specific systems, GPS-denied localization has also been advanced through broader state estimation pipelines such as VINS-Mono and LIO-SAM, which emphasize tightly coupled visual–inertial or lidar–inertial fusion for long-horizon drift control and map consistency [12,13]. In parallel, navigation in unstructured terrain has been explored through alternative planning families including artificial-potential-field methods, sampling-based planners, and other mobile robot path-planning frameworks beyond the frontier/A* combination adopted here [14,15]. These studies broaden the design space considered for GPS-denied autonomy. In the present work, frontier exploration, graph search, and PPO-based adaptation were selected as a lightweight and implementation-oriented combination for embedded SAR operation rather than as an exhaustive treatment of all planning strategies. The present work instead focuses on a single ground robot featuring Thermal–Visual Odometry (TV–VO), RescueGraph fusion, and beaconless return, achieving tightly coupled multi-sensor integration at low cost for GPS-denied autonomy. Unlike prior UAV or multi-robot studies, it prioritizes embedded efficiency and fully autonomous operation on a single deployable platform. Its remaining limitation is the lack of long-duration field validation, which is identified as a key next step for future work.

## 3. Methodology and System Design

Search and rescue (SAR) missions in collapsed or smoke-obscured environments suffer from unreliable Global Navigation Satellite System (GNSS) signals, making conventional localization infeasible. To enable autonomous operation under such constraints, the proposed ground robot integrates visual, thermal, inertial, and audio sensing into a unified low-cost autonomy stack.

Sensor-Centric Motivation:

The sensing design directly targets three dominant failure modes in GPS-denied disasters: (i) low texture or smoke-degraded RGB imagery; (ii) low-frame-rate thermal streams; (iii) intermittent standstill phases. Thermal gradients preserve correspondence under poor illumination, while IMU-driven ZUPT events suppress scale drift during micro-stands. Consequently, the system fuses Thermal+RGB+IMU within a Thermal–Visual Odometry (TV–VO) front end and exposes modality ablations (thermal-only, RGB-only, RGB+IMU, fusion) to quantify the contribution of each sensor. The full autonomous pipeline combines TV–VO for drift-resistant motion estimation, RescueGraph for probabilistic survivor detection, and loop-closure-based place recognition for beaconless return, achieving robust, GNSS-free navigation in confined disaster environments [16,17,18,19,20,21].

### 3.1. Dataset and Materials

Two main data sources were used: (i) synthetic and bench-generated sequences from a custom simulation scaffold providing grayscale frames, low-resolution thermal maps, IMU drift traces, and scalar audio activity signals; and (ii) simulation sequences emulating corridor and rubble environments. These controlled datasets enable validation of TV–VO and RescueGraph modules without physical trials. Ground truth from tape-measured landmarks or AprilTags is used only for offline evaluation. Optional CO_2_ and UWB sensors are planned for future extensions.

**Figure 1:**
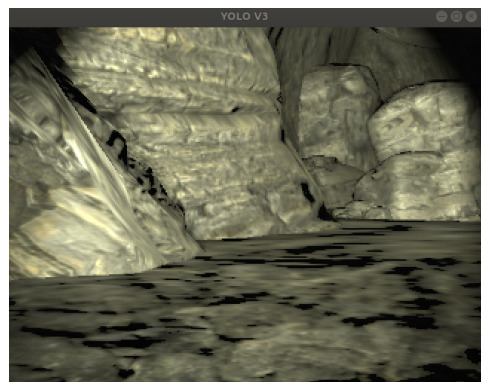
Subterranean tunnel scene from the CERBERUS SubT dataset, illustrating the low light and GPS-denied conditions targeted by the proposed system.

The CERBERUS SubT dataset [22], as illustrated in Figure 1, captures typical subterranean scenes with poor illumination, clutter, and conditions of total GPS loss motivating thermal–visual fusion for resilient localization.

#### 3.1.1. Sensor Calibration and TF-Based Frame Binding (Thermal–T265)

To enable metric-consistent spatial alignment between the thermal camera (FLIR Boson) and the Intel RealSense T265, we reconstructed the rigid-body extrinsics from the ROS TF graph recorded in the dataset. Specifically, TF relations were extracted from the /tf_static topic (available in Sensor_set_1), while camera intrinsics were extracted from camera_info (available in Sensor_set_2). This reflects the dataset organization: static inter-frame geometry is stored in one set, whereas intrinsic calibration parameters are stored in another.

##### Frames and Notation

Let $F_{b}$ denote the thermal camera link frame (boson_camera), $F_{base}$ the platform base frame (base_link), and $F_{t}$ the T265 reference frame (t265_link). A rigid transform from frame $F_{A}$ to $F_{B}$ is represented by a homogeneous matrix

(1) TAB=RABtAB0⊤1∈SE(3),

where RAB∈SO(3) is the rotation matrix and tAB∈R3 is the translation vector (meters).

##### TF Extraction and Composition

From /tf_static, we extracted the static TF edges that attach both sensors to the same platform base frame, namely base_link → boson_camera and base_link → t265_link. The desired extrinsics from the thermal frame to the T265 frame are obtained by composition through the shared base:

(2) Tbt=TbasetTbbase.

Since the dataset provides Ttbase and Tbbase in /tf_static, we compute

(3) Tbt=Ttbase−1Tbbase.

##### Estimated Extrinsics (Boson → T265)

Using the TF graph in Sensor_set_1, the final rigid transform from boson_camera to t265_link is as follows:

(4) tbt=0.0751050.1571040.19325m,Rbt=I3×3.

Thus, the homogeneous extrinsic matrix is

(5) Tbt=1000.0751050100.1571040010.193250001.

##### Practical Interpretation and Dataset Constraints

This transform binds the thermal sensor frame and the T265 reference frame into a single consistent TF tree, enabling downstream fusion tasks (e.g., associating thermal observations with motion/pose from T265). While the dataset includes T265 internal optical frame relations (e.g., t265_link → t265_fisheye*_frame →t265_fisheye*_optical_frame) in /tf_static, it does not provide an explicit thermal optical frame transform (e.g., boson_camera_optical_frame) or a dedicated thermal–T265 optical extrinsic. Therefore, we align at the link-frame level (boson_camera → t265_link) as a reproducible baseline derived purely from recorded TF, avoiding assumptions not supported by the bag content.

Accordingly, the thermal stream is used in this work as an auxiliary refinement cue rather than as a fully pixel-registered geometric measurement. The absence of a dedicated thermal-T265 optical extrinsic may reduce spatial precision near fine boundaries or under viewpoint change, but it does not replace the primary visual–inertial estimate. A full pixel-level thermal–visual calibration is left for future refinement.

##### Intrinsics (Used for Projection Where Applicable)

For completeness, intrinsic calibration parameters were extracted from camera_info messages in Sensor_set_2 (camera matrix K, distortion coefficients, and projection matrix P). These intrinsics are used for pixel-level projection/undistortion operations, while the TF-derived extrinsics above define the rigid spatial relationship between sensors.

### 3.2. Data Processing and Model Design

Visual frames are denoised using median or bilateral filters and downsampled for efficiency. Thermal images are normalized and temporally smoothed, while IMU readings are debiased via rolling mean correction with Zero-Velocity Updates (ZUPT). In the present implementation, ZUPT is used conservatively as a short-horizon drift control mechanism during near-stationary intervals. It is not intended as a full semantic classifier of robot intent or mobility failure and therefore does not by itself fully distinguish a deliberate sensing pause from entrapment, wheel slip, or high-centering on rubble. Audio signals are converted into energy envelopes (200–500 ms windows) for activity detection.

#### 3.2.1. Thermal–Visual Odometry (TV–VO)

Motion estimation proceeds in two stages; visual features (e.g., Oriented FAST and Rotated BRIEF (ORB) keypoints) provide coarse pose updates refined using gyroscope and thermal-edge cues. We maintain a planar pose $x_{t}\in \mathrm{SE}\left(2\right)$. A coarse visual increment $\Delta {\hat{x}}_{t}^{vo}$ is first estimated from visual features, then refined by an additive correction $\delta x_{t}$ obtained from IMU yaw and thermal edge alignment (Section 3.2.2). The global pose is updated via SE(2) composition.

Unlike optical flow TPL SLAM [5] or LiDAR thermal fusion [6], TV–VO keeps the refinement lightweight by aligning tracked thermal edge points through a distance-transform residual, which is suitable for low-cost sensing and embedded computation [23].

#### 3.2.2. Thermal Edge Refinement as a Robust Optimization Step

The TV–VO pipeline estimates a visual increment $\Delta {\hat{x}}_{t}^{vo}$ and then applies a correction that fuses (i) a yaw prior from IMU gyroscope integration and (ii) a thermal edge alignment constraint. We explicitly define the thermal refinement operator Δ(·) in Equation (6) as the solution of a robust least squares problem in the planar state space SE(2) with $x_{t}={\left[x_{t},y_{t},\theta_{t}\right]}^{T}$.

##### Thermal Edge Representation

Given the thermal image $T_{t}$, we extract a binary edge map $E_{t}=\mathrm{Edge}\left(T_{t}\right)$ using a fixed detector (Canny) and compute its Euclidean distance transform $D_{t}\left(\;\cdot \;\right)$:

(6) $$D_{t}\left(u\right)\;=\;\min_{v\;:\;E_{t}\left(v\right)=1}{∥u-v∥}_{2},$$

where $u,v\in R^{2}$ denote pixel coordinates. The distance transform enables a smooth edge alignment residual (zero when the point lies on a thermal edge).

##### Edge Points and Tracking

We sample *N* edge points from the previous thermal frame, $P_{t-1}={\left\{u_{i}\right\}}_{i=1}^{N}$ with $u_{i}$ chosen from pixels where $E_{t-1}=1$ (uniform sampling with non-maximum suppression). For each $u_{i}$, we obtain a tracked correspondence $v_{i}$ in $T_{t}$ using pyramidal Lucas–Kanade (LK) tracking on a fixed patch size (with forward–backward check to reject failures). The tracked set is denoted $C_{t}={\left\{\left(u_{i},v_{i}\right)\right\}}_{i=1}^{M}$ after outlier rejection (M≤N).

##### Correction Parameterization

Let $\delta x_{t}={\left[\delta x,\delta y,\delta \theta \right]}^{T}$ be a small correction added to the visual increment:

(7) $$\Delta x_{t}\;=\;\Delta {\hat{x}}_{t}^{vo}\;+\;\delta x_{t}.$$

We incorporate the gyroscope yaw increment $\Delta \theta_{t}^{imu}$ as a soft prior on the corrected yaw increment.

##### Thermal Residual and Robust Objective

For each tracked point $v_{i}$, the thermal alignment residual is defined using the distance transform:

(8) $$r_{i}^{th}\left(\delta x_{t}\right)\;=\;D_{t}\;\left(w(v_{i},\delta x_{t})\right),$$

where w(·) denotes a local first-order image-space warp parameterized by $\delta x_{t}$ under a small-motion approximation around $v_{i}$, implemented numerically in the solver. The correction is obtained by minimizing the following.

Let $\Delta {\hat{\theta }}_{t}$ denote the yaw component of the coarse visual increment $\Delta {\hat{x}}_{t}^{vo}$.

(9) $$\delta x_{t}^{*}=\arg\min_{\delta x_{t}}\sum_{i=1}^{M}\rho \;\left(\frac{r_{i}^{\mathrm{th}}\left(\delta x_{t}\right)}{\sigma_{\mathrm{th}}}\right)+\lambda_{\theta }\rho \;\left(\frac{\Delta {\hat{\theta }}_{t}-\Delta \theta_{t}^{\mathrm{imu}}}{\sigma_{\theta }}\right)+\lambda_{xy}\rho \;\left(\frac{{∥\;\left[\delta x,\delta y\right]}^{⊤}{\;∥}_{2}}{\sigma_{xy}}\right).$$

where ρ(·) is a robust loss (Huber), $\sigma_{th}$ scales pixel-distance residuals, and $\sigma_{\theta },\sigma_{xy}$ are normalization constants. $\lambda_{\theta }$ controls trust in the gyroscope yaw, while $\lambda_{xy}$ regularizes translation corrections to prevent drift due to spurious edges.

##### Solver and Implementation Details

We solve Equation (9) with Gauss–Newton (Levenberg–Marquardt optional) for a fixed small number of iterations (3–5) initialized at $\delta x_{t}=0$. Jacobians of $r_{i}^{th}\left(\;\cdot \;\right)$ are computed using finite differences on the warp w(·) and the distance transform $D_{t}\left(\;\cdot \;\right)$. Points with $D_{t}\left(\;\cdot \;\right)>\tau_{out}$ are discarded as outliers. The final TV–VO increment is therefore

(10) $$\Delta x_{t}\;=\;\Delta {\hat{x}}_{t}^{vo}\;+\;\delta x_{t}^{*}$$

which is composed into the global pose update using SE(2) composition.

For reproducibility and consistency across experiments, all front-end, refinement, and evaluation parameters are fixed and summarized in Table 2.

The blend weight α=0.6 is treated here as a fixed operating point selected for implementation simplicity and reproducibility under embedded constraints. We do not claim that this value is optimal across all SAR visibility regimes. In rapidly changing smoke, illumination, or thermal conditions, a confidence-adaptive weighting strategy based on feature quality or modality reliability would be a natural extension.

#### 3.2.3. Evidence Fusion (RescueGraph)

Thermal, motion, and audio cues are fused into a weighted score as follows:

(11) $$S_{t}=w_{\mathrm{th}}c_{\mathrm{th}}+w_{\mathrm{mot}}m_{t}+w_{\mathrm{aud}}a_{t},\;w_{\mathrm{th}}+w_{\mathrm{mot}}+w_{\mathrm{aud}}=1.$$

Here, thermal confidence $c_{th}$ denotes a normalized hotspot confidence derived from thermal intensity prominence within the candidate region, motion score $m_{t}$ denotes a normalized measure of frame-to-frame motion saliency around the same region, and audio energy $a_{t}$ denotes the short-window normalized acoustic envelope extracted from the microphone stream. Before weighted fusion, all three quantities are scaled to comparable numeric ranges so that no single modality dominates purely because of units.

Thresholds are set to $\tau_{1}=0.4$ and $\tau_{2}=0.7$ to define states, i.e., verification for $S_{t}>\tau_{1}$ and navigation commitment for $S_{t}>\tau_{2}$. [20,24].

#### 3.2.4. Mapping and Filtering

TV–VO and IMU poses are fused via an Extended Kalman Filter (EKF). The environment is represented as a log-odds occupancy grid updated via the observation model in Section 3.2.5.

#### 3.2.5. Mapping Observation Model and Bounded Coverage Metrics

##### Two-Dimensional Occupancy-Grid Observation Model (LiDAR-Free)

We maintain a 2D log-odds grid and update cells using a pose-driven visibility model derived from the sensing field-of-view (FoV) and range limits. At each time step *t*, the observation $z_{t}$ is defined as the set of FoV rays cast from the current pose $x_{t}$ within the camera frustum (horizontal/vertical FoV) up to a maximum range *R*. Each ray marks traversed cells as free space and marks its terminal cell as occupied (a conservative endpoint model), yielding an inverse sensor update $p(m_{i}|z_{t},x_{t})$ used in the standard log-odds recursion:

(12) $$l_{t}\left(m_{i}\right)=l_{t-1}\left(m_{i}\right)+\log\frac{p(m_{i}|z_{t},x_{t})}{1-p(m_{i}|z_{t},x_{t})}-l_{0}.$$

This model does not claim true geometric occupancy reconstruction (no depth/LiDAR); rather, it provides a consistent, repeatable visibility-to-grid projection suitable for SAR exploration under limited sensing. Because the map update is LiDAR-free and FoV-driven, thin obstacles such as wires, narrow protrusions, or small hanging debris may be weakly resolved or missed, especially under low-contrast conditions or unfavorable viewpoints. The resulting grid should therefore be interpreted as a conservative traversability approximation rather than a complete hazard sensor.

##### Bounded Two-Dimensional Coverage and Spillover (Fixing >100% Ambiguity)

To prevent inflated or non-standard “coverage >100%” interpretations, we report ROI-normalized map footprint inside a fixed region of interest (ROI) R derived from the annotated layout bounding box (with a small margin):

(13) $$\begin{array}{c} \mathrm{Coverage}@\mathrm{ROI}\left(\%\right)=100\;\cdot \;\frac{\left|O_{\mathrm{est}}\cap R\right|}{\left|R\right|},\\ \mathrm{Spillover}\left(\%\right)=100\;\cdot \;\frac{\left|O_{\mathrm{est}}\setminus R\right|}{\left|R\right|}, \end{array}$$

where $O_{\mathrm{est}}$ is the set of predicted occupied grid cells. Map agreement with the reference layout is additionally reported using IoU:

(14) $$\mathrm{IoU}=\frac{\left|O_{\mathrm{est}}\cap O_{\mathrm{gt}}\right|}{\left|O_{\mathrm{est}}\cup O_{\mathrm{gt}}\right|}.$$

These definitions are bounded and reproducible by construction.

Exploration uses frontier-based proposals with global A* and local DWA planning [21,25].

The 3D FoV voxel footprint is accumulated by union of per-pose ray-cast sets and normalized by the trajectory bounding box ROI; full details and results are reported in Section 7.5.

#### 3.2.6. Beaconless Return

Place recognition via bag-of-words (BoW) or keyframe similarity establishes loop closures linking the current position to the home base, enabling GPS free return [16,21]. In this work, BoW-based place recognition is used as a lightweight return aid rather than as a guarantee of relocalization given severe post-entry scene change. Secondary collapse, fire growth, or strong appearance shifts may reduce descriptor stability; therefore, return performance should be interpreted as depending on both appearance-based loop cues and the accumulated traversal structure, with stronger change-aware relocalization left for future work. Together, these modules form the complete GPS-denied SAR autonomy stack evaluated in Section 7.

## 4. System Architecture

The autonomy stack consists of four tightly coupled layers. Multimodal streams are first denoised and synchronized then fused in a unified perception pipeline for drift-resistant localization, robust detection, and real-time navigation [16,20,21].

### 4.1. Sensing

The robot employs low-cost sensors suitable for GPS-denied SAR missions: an RGB/Mono Camera for visual odometry and mapping, a Thermal Camera for LWIR perception through smoke or darkness, IMU for motion stabilization, and a Microphone for acoustic activity cues [20,24].

TF chain (static):

base_link→boson_camera,base_link→t265_link

Composition:

Tbt=Ttbase−1Tbbase

### 4.2. Pre-Processing

Raw inputs are normalized and time-aligned. Visual frames undergo denoising and keypoint extraction; thermal images are background-suppressed; IMU streams are debiased via ZUPT; and audio signals are converted into short time energy envelopes for fusion in RescueGraph.

### 4.3. Perception and Localization

Thermal, visual, and inertial cues are fused for continuous pose estimation. TV–VO combines ORB features, thermal edges, and IMU priors for drift-aware odometry, while an EKF maintains a consistent global pose. Loop closure further suppresses long-term drift in revisited areas [16,21].

### 4.4. Mapping and Detection

EKF poses populate a 0.1–0.2 m occupancy grid. Thermal hotspots and motion cues are fused by RescueGraph into a confidence score $S_{t}$, reducing false positives under degraded visibility [20,24].

### 4.5. Planning and Control

**Figure 2:**
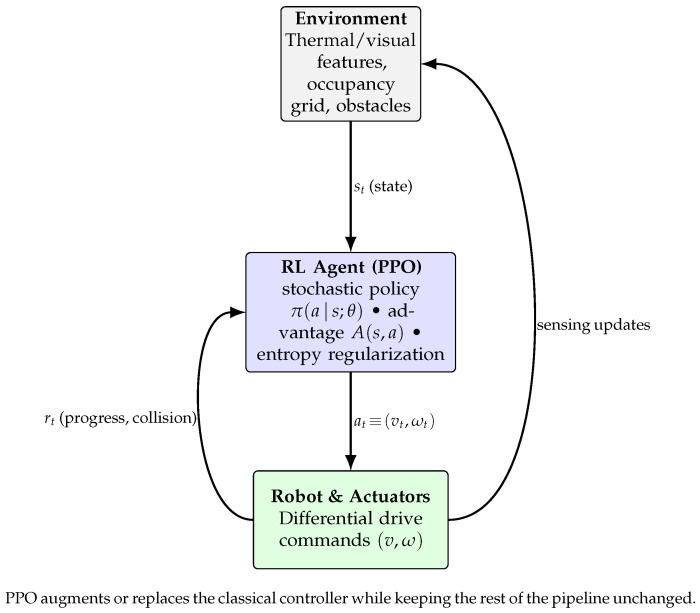
PPO-based reinforcement learning agent for adaptive navigation.

Frontier-based exploration and A* planning generate paths, while local avoidance ensures safe execution. The controller outputs smooth (v,w) commands, optionally replaced or assisted by a PPO agent that learns adaptive behavior from local maps and EKF poses (Figure 2). To clarify the system-level integration, the module-to-module dataflow-covering outputs, downstream consumers, and their functional roles is summarized in Table 3.

### 4.6. Communication and User Interface

A lightweight mesh network relays RescueGraph alerts, pose updates, and planner logs across multiple hops, preserving connectivity in GPS-denied or obstructed environments. Nodes act as relays for automatic re-routing and redundant forwarding under link degradation or failure. The operator monitors live maps, robot pose, and frontier progress through a graphical UI with optional real-time override [7].

#### 4.6.1. Connection to Ablation Studies

Communication is functionally decoupled from sensing, mapping, and control, allowing individual modules to be disabled for ablation. Removing thermal or audio inputs, for instance, directly quantifies their contribution to localization and detection, as analyzed in Section 8.

##### Dataflow Overview

**Figure 3:**
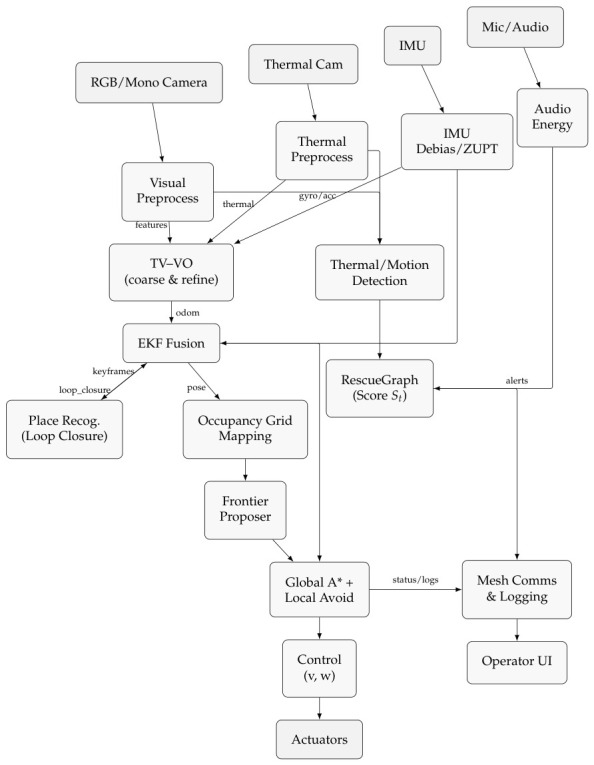
System architecture with sensing, perception/localization, mapping, detection, planning/control, and comms/logging.

RGB, thermal, IMU, and audio streams are pre-processed, then fused through *TV–VO* and *EKF* to generate drift aware poses for mapping and detection. RescueGraph combines thermal and motion cues into an alert score $S_{t}$, while frontier selection drives global A* planning and local avoidance. The controller issues (v,w) commands to the actuators, and the mesh layer forwards alerts and logs to the operator for continuous supervision (Figure 3).

Algorithm 1 (TV–VO), Algorithm 2 (RescueGraph), and Algorithm 3 (mission state machine) summarize the core computation pipeline [21,24].

**Algorithm 1** Thermal–Visual Odometry (TV–VO)
**Require:** Visual frame $I_{t}$, thermal map $T_{t}$, IMU packet $I_{t}$, previous pose ${\hat{x}}_{t-1}$**Ensure:** Updated pose estimate ${\hat{x}}_{t}$  1:Pre-process $I_{t},T_{t}$ by denoising and normalizing intensities  2:$(u_{t},\Sigma_{u})\leftarrow$ estimate visual odometry increment from $I_{t}$  3:Δθ← integrate gyroscope rotation from $I_{t}$  4:$\delta x_{t}^{*}\leftarrow \arg\min_{\delta x_{t}}\;J\left(\delta x_{t}\right)$ using Equation (9) (thermal edge alignment + IMU yaw prior).  5:

$\Delta x_{t}\leftarrow \Delta {\hat{x}}_{t}^{vo}+\delta x_{t}^{*}$

  6:

${\hat{x}}_{t}\leftarrow {\hat{x}}_{t-1}⊕\Delta x_{t}$

  7:**return**${\hat{x}}_{t}$                          ▹ drift-reduced pose estimateNote:⊕ denotes SE(2) pose composition.


**Algorithm 2** RescueGraph: Multimodal Evidence Fusion
**Require:** Thermal confidence $c_{\mathrm{th}}$, audio energy $a_{t}$, motion score $m_{t}$, and adaptive weights $w_{\mathrm{th}},w_{\mathrm{aud}},w_{\mathrm{mot}}$**Ensure:** Computed fusion score $S_{t}$  1:

$S_{t}\leftarrow w_{\mathrm{th}}c_{\mathrm{th}}+w_{\mathrm{aud}}a_{t}+w_{\mathrm{mot}}m_{t}$

  2:**if** $S_{t}>\tau_{2}$ **then**  3:    Commit to assist operation (strong evidence)  4:**else if** $S_{t}>\tau_{1}$ **then**  5:    Trigger verification of the candidate region  6:
**else**
  7:    Continue exploration (no significant evidence)  8:
**end if**
  9:**return** $S_{t}$                         ▹ Fused multimodal confidence score


**Algorithm 3** Mission State Machine (SM)
1:**while** mission not complete **do**2:    Update *TV–VO*, *EKF*, and *map modules*3:    $S_{t}\leftarrow$ RescueGraph(·)4:    **if** $S_{t}\leq \tau_{1}$ **then**5:        path ← A*(pose, frontier)                 ▹ continue exploration6:    **else if** $\tau_{1}<S_{t}\leq \tau_{2}$ **then**7:        path ← A*(pose, region-of-interest)           ▹ verify potential survivor8:    **else**9:        Broadcast alert; path ← A*(pose, victim location)         ▹ assist operation10:    **end if**11:    cmd ← LocalAvoidance(path)12:    Apply(cmd)                      ▹ execute motion command13:    **if** place-recognition closure or mission end **then**14:        path ← A*(pose, home)                      ▹ return-to-base15:    **end if**16:
**end while**



##### Example Scenario

In a smoke-filled corridor, *TV–VO* maintains odometry while RescueGraph detects a thermal hotspot with audio peaks. When $S_{t}>\tau_{2}$, the robot approaches, triggers a mesh alert, and returns via loop closure.

### 4.7. Output Collection and Analysis

The system logs sensor streams, poses, and maps for offline evaluation using five metric groups: **Localization** (ATE, RPE), **Mapping** (coverage, grid accuracy), **Detection** (precision, recall, F1, ROC), **Planning/Control** (path length, detection time, success rate), and **Runtime** (latency, CPU load) on embedded hardware.

### 4.8. Complexity Analysis

Let $n_{f}$, $n_{c}$, *N*, and *E* denote visual features, thermal blobs, grid cells, and graph edges. TV–VO: $O(n_{f}\log n_{f})$ for matching, $O(n_{f}+n_{c})$ for refinement. EKF: O(1) per update. Mapping: O(k) for k≪N updated cells. RescueGraph: O(1) per frame. Planning: O(N) for frontier detection, O(ElogV) for A*, and O(1) for local avoidance. Runtime is dominated by feature tracking and grid search, both of which are feasible in real time on embedded platforms [21,25].

## 5. Practical Implementation and Field Readiness

### 5.1. Practical Feasibility and GPS-Denied Justification

The stack in Figure 3 runs in real time (10–20 Hz) on a compact SBC (ARM or x86), executing TV–VO, IMU fusion, mapping, and detection without heavy neural models. Sensors include a global shutter RGB/mono camera, a low-resolution LWIR module (80 × 60 or 160 × 120), a 6-DoF IMU (≥100 Hz), and a 16 kHz MEMS microphone, enabling operation in smoke, dust, and low light [22,24]. Global A* and local avoidance handle path planning, while the controller outputs smooth (v,w) commands for differential drive [21,25].

#### 5.1.1. Suitability for Confined and Rubble Environments

Thermal sensing supports perception in darkness or smoke; IMU priors stabilize motion under visual loss; audio energy provides a low-cost human cue. A compact chassis and narrow sensor layout allow traversal in tight corridors, and bench/simulation tests emulate debris occlusion and sensor dropouts to validate robustness [22,24].

#### 5.1.2. Simulation of Collapse Conditions

Simulations model light attenuation, thermal occlusion, RF interference, and IMU vibration noise. Metrics include ATE/RPE for localization, coverage and occupancy accuracy for mapping, and precision/recall/F1 of $S_{t}$ for detection. Ablation studies quantify each module’s contribution under partial degradation [22,24].

#### 5.1.3. Why GPS-Denied?

Urban SAR missions take place in reinforced structures, tunnels, and basements where GNSS signals are blocked or distorted by concrete and steel. Multiple paths and limited sky view make satellite fixes unreliable.

This infrastructure-free design avoids dependence on GNSS and external localization aids, relying instead on TV–VO, IMU priors, loop closure, and onboard planning. Under the evaluated conditions, this supports continued navigation even when external aids are unavailable, although performance still depends on sensing quality and place recognition reliability [7,22,26]. The required hardware components and their correspondence to simulated modules are summarized in Table 4.

### 5.2. Disaster-Ready Mesh Networking: Design and Deployment

#### 5.2.1. Why Mesh Networking for Disasters?

Disaster scenarios often destroy traditional Wi-Fi or cellular infrastructure. A self-healing peer-to-peer mesh network preserves connectivity by relaying packets across multiple hops without centralized coordination; it is well suited for dynamic SAR missions where robots, responders, and beacons are constantly in motion. Even with partial links, critical RescueGraph alerts and planner logs remain deliverable [7,22].

#### 5.2.2. Mesh Properties, Protocols, and Deployment

The system uses dual-band radios: 2.4/5 GHz for short range telemetry and an optional sub-GHz backbone (868/915 MHz) for long-range penetration. Compact COTS modules exchange small, prioritized packets alerts, pose updates, and map tiles using buffering and lightweight encryption for robustness and privacy [22,29]. Deployment is fully GPS-independent: a portable gateway at the entry point and relay “breadcrumbs” dropped along corridors or stairwells create a vertical multi-hop topology. As the robot moves deeper, it autonomously re-routes around obstacles, sustaining a persistent link to the operator even in basements or rubble voids [26].

#### 5.2.3. Coverage and Engineering Considerations

Typical 2.4 GHz hops span 20–40 m indoors, while sub-GHz links reach 80–150 m. Chains of two–five relays extend coverage across multiple rooms or floors. Rather than using high-power transmitters, range scales linearly with relay count *N* and spacing *d*:

(15) $$D_{\mathrm{total}}\approx \alpha Nd,\;\alpha \approx 0.8.$$

Practical measures such as additional relays, mixed-frequency bands, higher gain antennas, adaptive data rates, and autonomous relay placement extend total coverage from tens to hundreds of meters [22,26]. Representative per-hop communication ranges and recommended relay spacing under different SAR conditions are summarized in Table 5.

#### 5.2.4. Cost and End-to-End Readiness

All radios and relays use compact, low-power commercial off-the-shelf (COTS) components operating for several hours on small batteries. The lightweight, modular software stack compresses and rate-limits data to conserve bandwidth, while GPS-free navigation removes the need for real-time kinematic (RTK) receivers or external beacons. Integrated as described, the communication and navigation design is intended to support extended-range missions with persistent mesh connectivity, subject to relay spacing, materials, and power conditions. These results should be interpreted as an implementation-oriented feasibility argument rather than as a completed large-scale field deployment benchmark [7,22,26].

**Figure 4:**
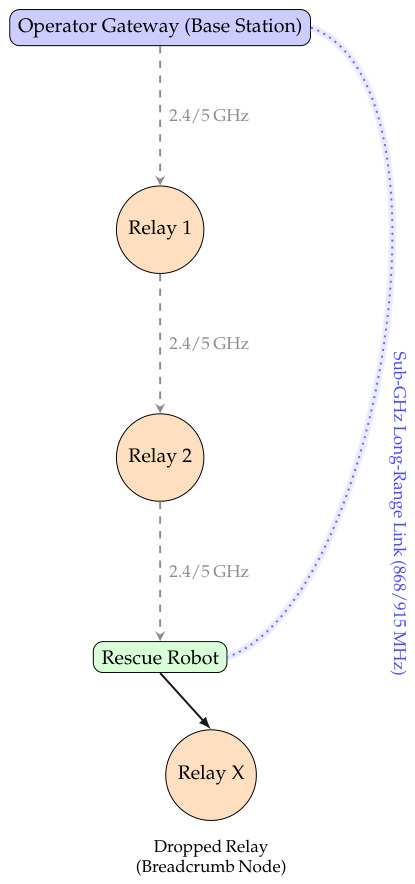
Dual-band multi-hop mesh network linking the operator gateway, relay nodes, and rescue robot in GPS-denied environments.

The dual-band multi-hop mesh communication architecture enabling persistent connectivity in GPS-denied environments is illustrated in Figure 4.

## 6. Experimental Setup

This section summarizes the environment, hardware, datasets, and evaluation protocols used to validate the GPS-denied search and rescue (SAR) autonomy stack.

### 6.1. Hardware Platform

Experiments were conducted on a ground robot equipped with the multi-sensor suite shown in Figure 3: a grayscale or RGB camera (10–15 fps) for odometry, an LWIR thermal camera (8–14 μm, 5–9 fps) for perception under smoke or darkness, a 6-DoF IMU for motion stabilization, and a MEMS microphone for energy-based audio detection in RescueGraph. All modules ran in real time on a quad-core ARM single-board computer (SBC) with 4 GB RAM, reflecting the low-power constraints of deployable SAR robots [22,28,29].

#### Implementation Note (Results/Replicability)

We reconstructed the thermal–T265 extrinsics directly from the recorded TF tree. Specifically, we parsed /tf_static (Sensor_set_1) to obtain the static edges base_link → boson_camera and base_link → t265_link, then composed them to compute Tbt=Ttbase−1Tbbase. The resulting translation was ${\left[0.075105,\;0.157104,\;0.19325\right]}^{⊤}$ m with Rbt=I, yielding a reproducible link–frame alignment for downstream fusion. Intrinsics required for pixel-level operations were read separately from camera_info (Sensor_set_2).

### 6.2. Datasets

Two dataset classes were employed: **(i) Synthetic/Bench Datasets** included custom grayscale video, thermal maps, IMU drift traces, and audio energy signals for isolated validation of TV–VO and RescueGraph, and **(ii) the Pilot-Run Datasets** included indoor corridor and mock-rubble logs with synchronized thermal, visual, IMU, and audio streams. Ground truth from AprilTag landmarks was used only for offline evaluation, not during runtime. Accordingly, the present evaluation should be interpreted as a proof-of-concept validation built from synthetic/bench data, pilot indoor logs, and benchmark-style analysis rather than as a completed real-disaster field deployment study. Controlled physical robot trials in larger and less structured SAR environments remain an important next-stage validation target.

### 6.3. Evaluation Protocols and Scenarios

Scenarios replicated key GPS-denied SAR conditions: **(i)** cluttered, low-visibility corridors for odometry and mapping; **(ii)** survivor detection using injected thermal and audio cues for testing RescueGraph thresholds $\left(\tau_{1},\tau_{2}\right)$; and **(iii)** return-to-base navigation via loop closure recognition without GNSS or beacons. Metrics followed Section 3 across localization, mapping, detection, and planning/control. Each test was repeated 3 times across 6 scenarios (18 total runs) for statistical consistency [7,22].

To avoid ambiguity across short indoor trials and long-range KITTI evaluations, Table 6 summarizes all error metrics, units, alignment modes, and reporting windows used throughout the paper.

Short indoor trials (Table 7) are evaluated with Sim(3) alignment and dense frame-to-frame ATE/RPE, yielding centimeter-scale errors due to limited drift accumulation. Conversely, long-range KITTI-08 (Table 9, Table 10 and Table 11) reports global APE/RPE over the full path, exposing accumulated drift (tens of meters). Thus, ATE (cm-scale) and APE (m–tens of m) differ by design rather than inconsistency.

**Table 7:** Internal localization accuracy (ATE/RPE) averaged over controlled runs.

Method	ATE [m]	RPE [m]
Visual-only VO	0.0577	0.0214
TV–VO (ours)	0.0263	0.0208
TV–VO + ZUPT	**0.0207**	**0.0207**

**Table 8:** Cross-dataset localization validation (ATE/RPE in meters).

Dataset	ATE [m]	RPE [m]
KITTI Sequence 00	0.0880	2.5147
KITTI Sequence 08	0.1024	3.8680
CERBERUS (No GT)	–	–

**Table 9:** Absolute Pose Error (APE) on KITTI (translation, meters). Lower is better. ORB-VO (orbpy) is a visual-only ORB feature-based baseline; TV-VO fuses thermal cues. TV-VO(inv) is an ablation (with thermal channel inverted) to test whether gains come from an informative thermal structure.

Method	RMSE	Mean	Median	Std
ORB-VO (orbpy)	77.5	66.7	51.9	39.3
TV-VO	38.1	33.2	28.6	18.7
TV-VO (inv-thermal)	154.0	137.0	130.5	70.4

**Table 10:** Relative Pose Error (RPE) on KITTI (translation, meters) within two evaluation windows. Δ=10 frames is the standard KITTI setting; Δ=1 is reported for short-horizon sensitivity. Lower is better.

Method	Δ	RMSE	Mean	Median	Std
ORB-VO (orbpy)	1	1.62	1.59	1.65	0.30
TV-VO	1	1.72	1.69	1.76	0.29
ORB-VO (orbpy)	10	16.0	15.6	16.5	3.59
TV-VO	10	14.5	14.1	14.9	3.35

**Table 11:** Relative Pose Error (RPE) on KITTI (rotation, degrees) with Δ=10 frames. Lower is better.

Method	RMSE	Mean	Median	Std
ORB-VO (orbpy)	31.2	13.4	3.09	28.2
TV-VO	17.04	9.01	2.58	14.5

**Table 12:** Detection performance of RescueGraph on PST900 dataset.

Dataset	Precision	Recall	F1	AUC
PST900 (ours)	1.000	0.615	0.762	–

**Table 13:** Detection performance on CERBERUS dataset.

Scenario	Precision	Recall	F1
RescueGraph (ours)	0.818	0.600	0.6923

ATE Definition: ATE is reported as the RMSE of per-frame Absolute Pose Error under Sim(3) Umeyama alignment (evo toolkit). Short-segment ATE (Table 7) reflects indoor simulation sequences of limited path length (≈48 m), while full-trajectory ATE (Table 8) reflects accumulated drift over the complete KITTI-08 path. The two values are not directly comparable due to the order-of-magnitude difference in trajectory length.

RPE Step: We use a fixed relative step Δ that is explicitly stated per dataset. For KITTI, we report Δ=10 frames; for indoor sequences, we report Δ=1 frame due to shorter trajectories and denser sampling. All RPE tables/figures follow this convention and specify Δ in their captions.

#### Trajectory Evaluation Metrics (ATE/RPE)

We evaluate localization accuracy in the SE(3) pose space using the standard Absolute Trajectory Error (ATE) and Relative Pose Error (RPE). Although our pose logs are 2D (x,y,θ), each record is lifted to an SE(3) transform by embedding yaw as a rotation about the *z*-axis and setting z=0. To ensure fair comparison under potential scale ambiguity, the estimated trajectory is aligned to the reference using Umeyama similarity alignment with scale:

(16) $$p_{i}^{\;\mathrm{gt}}\approx s\;R\;p_{i}^{\;\mathrm{est}}+t,$$

where (s,R,t) are obtained by minimizing the least squares error over all matched timestamps. We report ATE as the root mean square Euclidean position error (meters):

(17) $${\mathrm{ATE}}_{\mathrm{RMSE}}=\sqrt{\frac{1}{N}\sum_{i=1}^{N}{∥p_{i}^{\;\mathrm{est},\mathrm{aligned}}-p_{i}^{\;\mathrm{gt}}∥}_{2}^{2}},$$

and RPE over a fixed step Δ (see RPE step note above) using the relative motion:

(18) $$\Delta T_{i}={\left(T_{i}^{\mathrm{gt}}\right)}^{-1}{T_{i+\Delta }^{\mathrm{gt}}}^{\;-1}\;{\left(T_{i}^{\mathrm{est}}\right)}^{-1}T_{i+\Delta }^{\mathrm{est}}.$$

RPE translation is reported in meters as $∥\Delta t_{i}{∥}_{2}$ and RPE rotation in degrees using the SO(3) geodesic angle:

(19) $$\theta_{i}=\cos^{-1}\;\left(\frac{\mathrm{tr}(\Delta R_{i})-1}{2}\right).$$

The trajectory alignment and error computation procedure based on Umeyama similarity transformation is summarized in Algorithm 4.
**Algorithm 4** Trajectory Alignment and Error Computation (ATE/RPE, Umeyama with scale) **Require:** Ground truth poses ${\left\{T_{i}^{gt}\right\}}_{i=1}^{N}$, estimated poses ${\left\{T_{i}^{est}\right\}}_{i=1}^{N}$, step Δ **Ensure:** ${\mathrm{ATE}}_{RMSE}$, ${\mathrm{RPE}}_{trans}$, ${\mathrm{RPE}}_{rot}$     1: Extract positions $p_{i}^{gt}\leftarrow \mathrm{trans}\left(T_{i}^{gt}\right)$, $p_{i}^{est}\leftarrow \mathrm{trans}\left(T_{i}^{est}\right)$     2: (s,R,t)← UmeyamaAlignWithScale $\left(\left\{p_{i}^{est}\right\},\left\{p_{i}^{gt}\right\}\right)$     3: $p_{i}^{est,aligned}\leftarrow s\;R\;p_{i}^{est}+t$
▹ Sim(3) alignment    4: $e_{i}\leftarrow {∥p_{i}^{est,aligned}-p_{i}^{gt}∥}_{2}$     5: ${\mathrm{ATE}}_{RMSE}\leftarrow \sqrt{\frac{1}{N}\sum_{i=1}^{N}e_{i}^{2}}$     6: **for** i=1 to N−Δ **do**     7:       $\Delta T_{i}^{gt}\leftarrow {\left(T_{i}^{gt}\right)}^{-1}T_{i+\Delta }^{gt}$     8:       $\Delta T_{i}^{est}\leftarrow {\left(T_{i}^{est}\right)}^{-1}T_{i+\Delta }^{est}$     9:       $E_{i}\leftarrow {\left(\Delta T_{i}^{gt}\right)}^{-1}\Delta T_{i}^{est}$   10:       $e_{i}^{trans}\leftarrow {∥\mathrm{trans}(E_{i})∥}_{2}$   11:       $e_{i}^{rot}\leftarrow \cos^{-1}\;\left(\frac{\mathrm{tr}(\mathrm{rot}\left(E_{i}\right))-1}{2}\right)$   12: **end for**   13: ${\mathrm{RPE}}_{trans}\leftarrow \sqrt{\frac{1}{N-\Delta }\sum_{i=1}^{N-\Delta }{\left(e_{i}^{trans}\right)}^{2}}$   14: ${\mathrm{RPE}}_{rot}\leftarrow \frac{180}{\pi }\sqrt{\frac{1}{N-\Delta }\sum_{i=1}^{N-\Delta }{\left(e_{i}^{rot}\right)}^{2}}$
▹ degrees

## 7. Results and Analysis

The GPS-denied SAR autonomy stack was evaluated in terms of localization, mapping, detection, and runtime performance. All experiments were repeated three times, and average results are reported.

### 7.1. Internal Localization Accuracy

Table 7 summarizes the performance of the proposed Thermal–Visual Odometry (TV–VO) against a visual-only baseline. Fusing thermal–visual cues achieved centimeter-level drift reduction and smoother motion estimates.

#### Internal Localization Accuracy (Example Run)

Using Umeyama alignment with scale (Section Trajectory Evaluation Metrics (ATE/RPE)), the evaluated sequence (N=1200) achieved ${\mathrm{ATE}}_{\mathrm{RMSE}}=0.0201$ m, ${\mathrm{RPE}}_{\mathrm{trans}}=0.0205$ m, and ${\mathrm{RPE}}_{\mathrm{rot}}=0.792^{\circ }$ at Δ=1 frame, with an estimated scale factor s=1.000087.

For long-range sequences (e.g., KITTI), we report trajectory-level errors using the same Sim(3) alignment protocol and a fixed Δ window (Section Trajectory Evaluation Metrics (ATE/RPE)). Accordingly, the long-range evaluation emphasizes accumulated drift patterns over the full trajectory rather than short-term frame-to-frame consistency.

Overall, this internal benchmark demonstrates consistent behavior across controlled runs, supporting the practical feasibility of the proposed pipeline for embedded operation [22,24].

### 7.2. Cross-Dataset Validation

**Figure 5:**
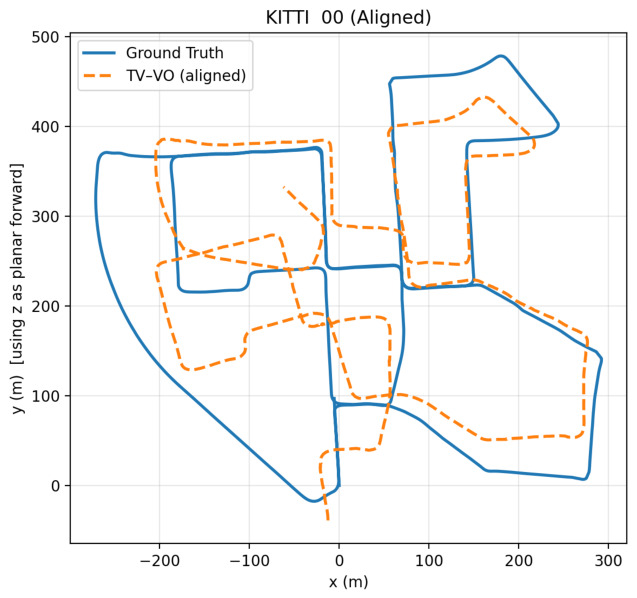
Aligned TV–VO trajectory on KITTI Sequence 00.

**Figure 6:**
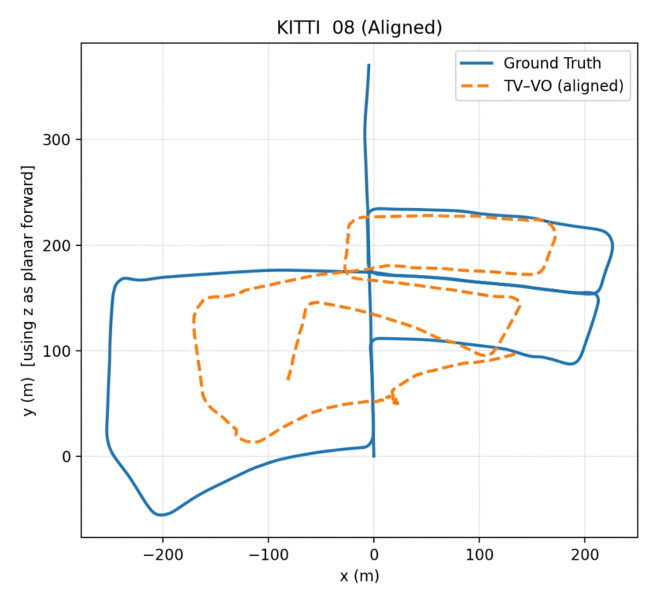
TV–VO trajectory on KITTI Sequence 08.

**Figure 7:**
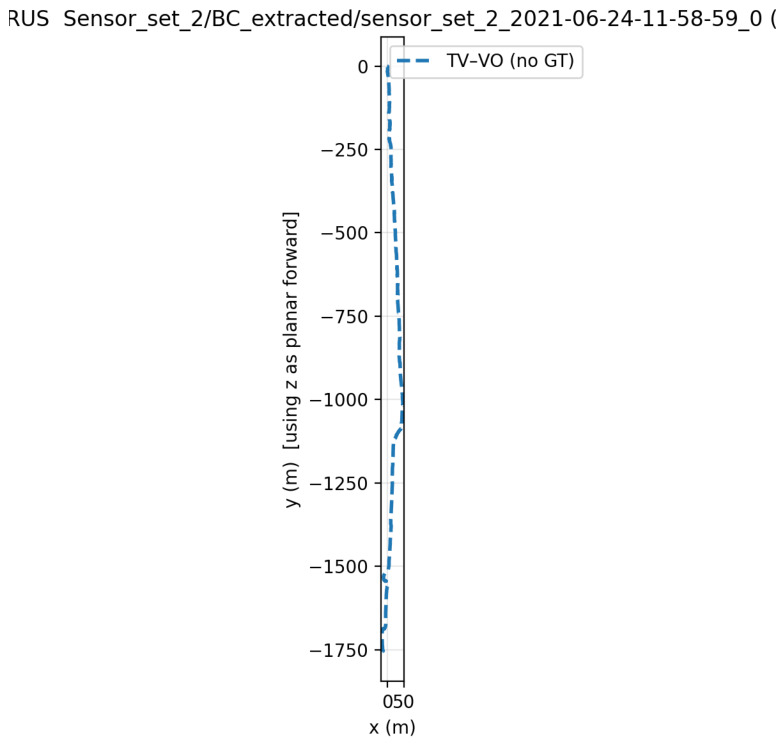
Qualitative trajectory on the CERBERUS Subterranean dataset.

TV–VO was also evaluated on KITTI (Seq. 00, 08) and CERBERUS datasets. The results in Table 8 and Figure 5, Figure 6 and Figure 7 show reduced pose errors on KITTI and stable subterranean trajectories on CERBERUS under GPS-denied conditions.

#### 7.2.1. External Comparison on KITTI

**Figure 8:**
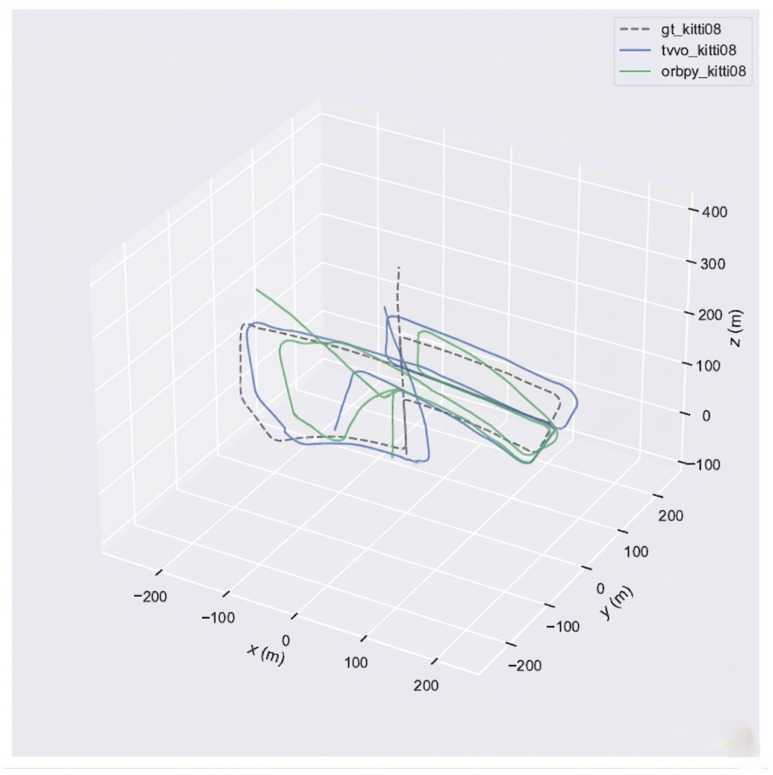
KITTI-08 trajectory overlay (GT vs. TV–VO vs. ORB–VO (orbpy)).

**Figure 9:**
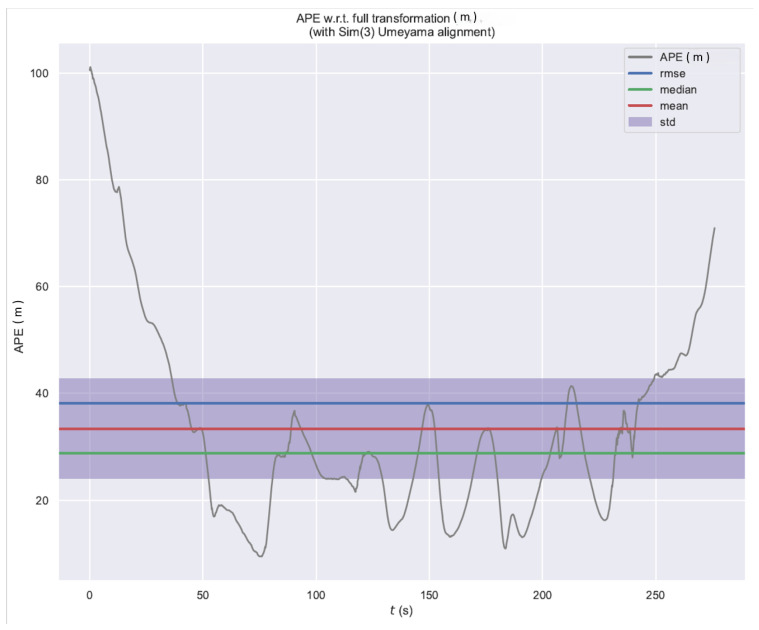
AbsolutePose Error (APE) of TV–VO over time on KITTI-08 (Sim(3) alignment).

**Figure 10:**
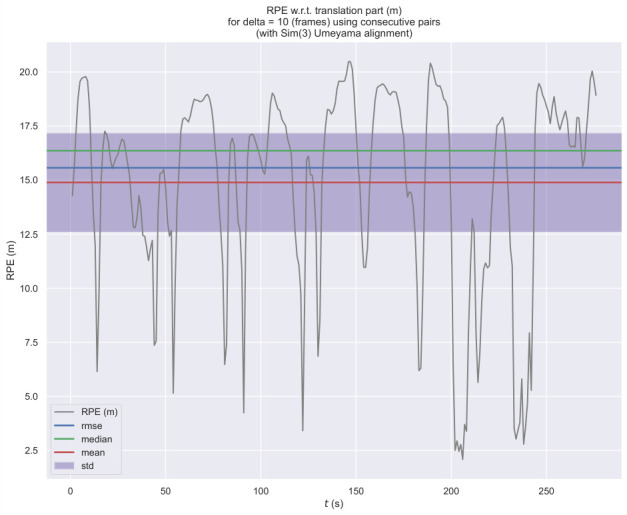
Relative Pose Error (RPE) of TV–VO (Δ=10 frames) on KITTI-08 (Sim(3) alignment).

Using the evo evaluation toolkit on KITTI-08, we compare TV–VO against a visual-only ORB feature-based baseline (ORB–Py) under the same calibration, trajectory format, and Sim(3) alignment. To make the baseline gap explicit and reproducible, we report Absolute Pose Error (APE) and Relative Pose Error (RPE) with a fixed window Δ (KITTI default: Δ=10 frames), separating translation and rotation components. Figure 8, Figure 9 and Figure 10 visualize the trajectory overlay and error distributions, confirming the quantitative trends in Table 9, Table 10 and Table 11. Notably, the short-horizon RPE (Δ=1) remains comparable, while Δ=10 highlights the reduction in accumulated drift achieved by thermal refinement.

Notably, the short-horizon RPE (Δ=1) remains comparable across methods, while the long-horizon window (Δ=10) highlights the main benefit of thermal refinement in reducing accumulated drift.

The Absolute Pose Error behavior is further illustrated in Figure 9.

#### 7.2.2. Trajectory and Error Patterns

**Figure 11:**
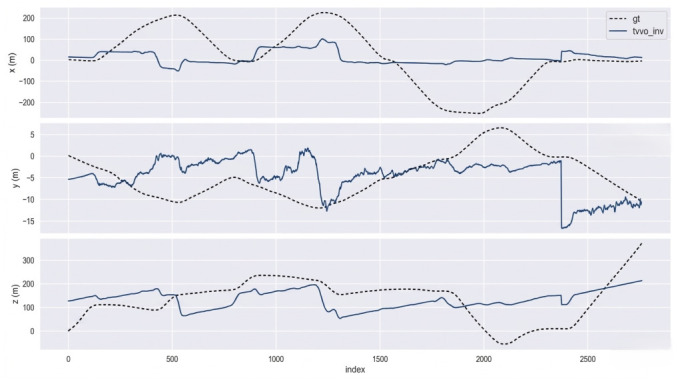
Comparative translational trajectories of TV–VO and KITTI ground truth.

**Figure 12:**
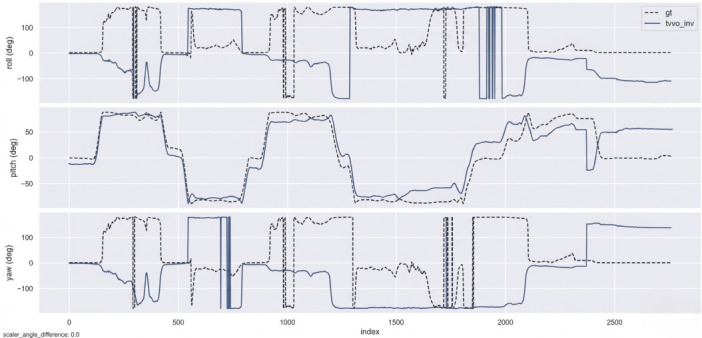
Rotational trajectories (roll, pitch, yaw) of TV–VO and KITTI ground truth.

**Figure 13:**
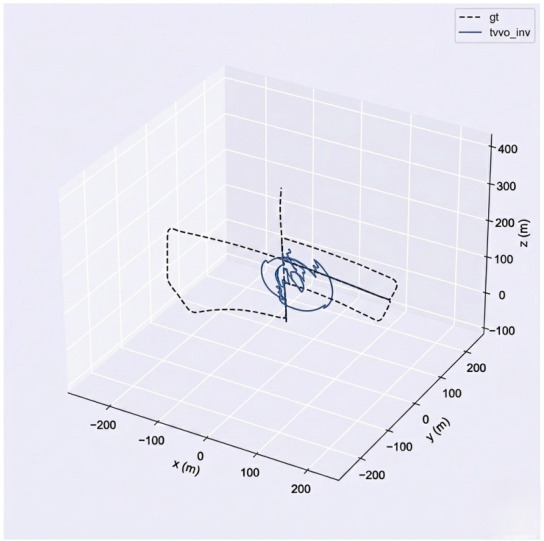
Three-dimensional overlay of TV–VO and ground truth illustrating coarse trajectory trend agreement under accumulated long-horizon drift.

Figure 9, Figure 11, Figure 12 and Figure 13 illustrate translational, rotational, and three-dimensional trajectory behavior relative to KITTI ground truth. These plots should be interpreted qualitatively: TV–VO follows several coarse turning trends, but noticeable deviations remain under full-trajectory evaluation because drift accumulates over long horizons. Accordingly, the external KITTI analysis is used here to assess relative drift behavior and baseline separation under a unified protocol, not to claim tight absolute agreement with ground truth throughout the sequence. This distinction is important when interpreting acceptability: the indoor pilot evaluations target short-horizon consistency relevant to local navigation and return behavior, whereas the KITTI-style analysis stresses accumulated drift over substantially longer trajectories. The latter therefore serves as a stress test of drift behavior rather than as evidence of centimeter-level global correspondence.

Overall, TV–VO shows consistent behavior under the present evaluation protocol and remains computationally lightweight; future work will benchmark against ORB SLAM2/DSO/VINS Mono under the same KITTI and CERBERUS protocols.

### 7.3. Detection Performance on CERBERUS

**Figure 14:**
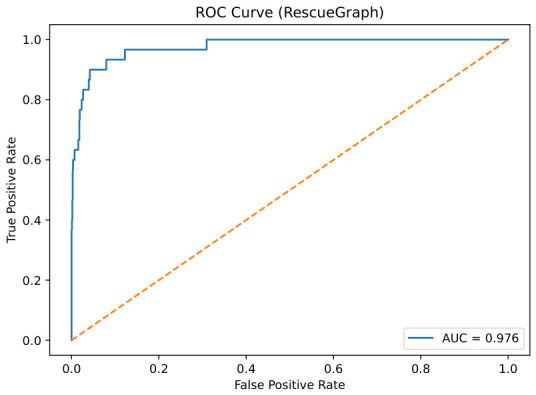
ROC curve of $S_{t}$ for survivor detection on CERBERUS (AUC = 0.976). The orange dotted line represents the random-classifier baseline.

The RescueGraph detector was assessed using precision/recall/F1. Table 13 shows precision 0.82 and recall 0.60 (F1 = 0.6923), and Figure 14 reports AUC = 0.976, confirming reliable fusion-driven discrimination. From an operational SAR perspective, this detector is better interpreted as a triage-oriented cueing module than as a precision-maximizing final classifier. The strong AUC indicates that the fused score ranks positive cases well across thresholds, whereas the more moderate F1 reflects threshold sensitivity under noisy multimodal overlap. In practice, likely audio failure cases include machinery noise, impact-like transients, enclosed-space reverberation, and weak or partially occluded human vocalizations, all of which can either mask valid distress activity or introduce spurious acoustic evidence.

### 7.4. Cross-Dataset Validation on PST900

**Figure 15:**
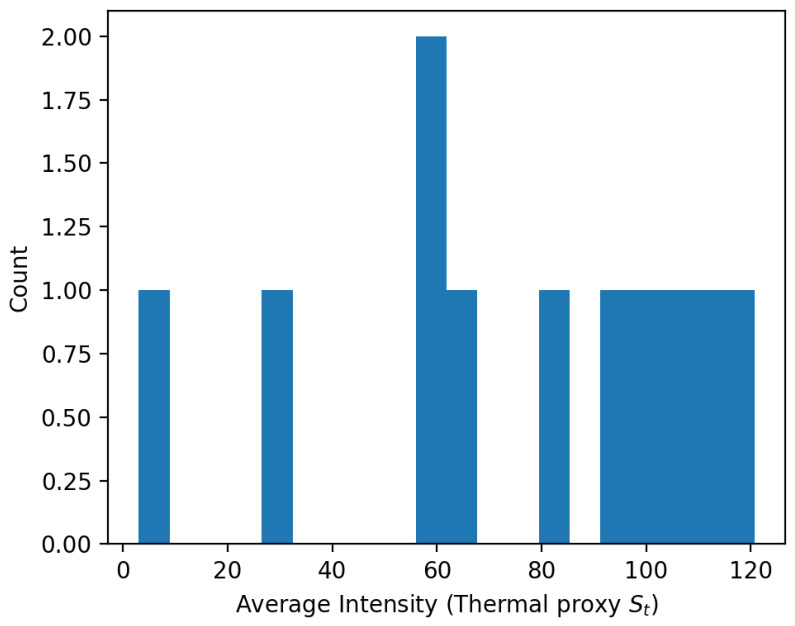
Thermal intensity distribution on PST900 frames showing stable detector activation.

On the PST900 thermal RGB benchmark, RescueGraph maintained precision = 1.0, recall = 0.62 (F1 = 0.76), as summarized in Table 12. stable thermal intensity distributions (Figure 15) indicate robust activation despite limited diversity. Localization results on KITTI and CERBERUS follow Table 8 (Section 7.2).

CERBERUS (field-like noise) favors conservative recall to avoid false alarms, while PST900 (clean images) yields perfect precision with some misses—evidence of domain-aware behavior suitable for SAR deployment.

### 7.5. Mapping Quality

#### 7.5.1. Two-Dimensional Occupancy Footprint (Bounded and ROI-Normalized)

Occupancy grid agreement against the annotated layout is evaluated using IoU and ROI-normalized footprint coverage. To avoid ambiguous “coverage >100%” interpretations, we define a fixed region of interest (ROI) from the ground truth layout bounding box (with a small margin) and report the following.

Coverage@ROI, Spillover, and IoU are computed as defined in Section 3.2.5, Equations (13) and (14). The previous “coverage >100%” values were caused by an unbounded denominator; we resolve this by normalizing coverage to a fixed ROI and explicitly reporting out-of-ROI mass via Spillover.

Under this corrected protocol, the corridor scenario achieves Coverage@ROI=1.3% with IoU=0.638 and Spillover=0.0%, indicating spatially consistent occupancy estimates without out-of-ROI expansion.

The ROI-bounded mapping performance is summarized in Table 14.

#### 7.5.2. 3D FoV Voxel Footprint (Ray-Casting; Not Occupancy)

To complement 2D occupancy evaluation, we report a lightweight 3D FoV voxel footprint metric based on deterministic ray-casting using the known sensing geometry (FoV and range), without depth reconstruction. Importantly, this is not a volumetric occupancy estimate (no LiDAR/stereo depth); rather, it is a geometry-consistent proxy of the space that could be observed along the traversed trajectory.

**Figure 16:**
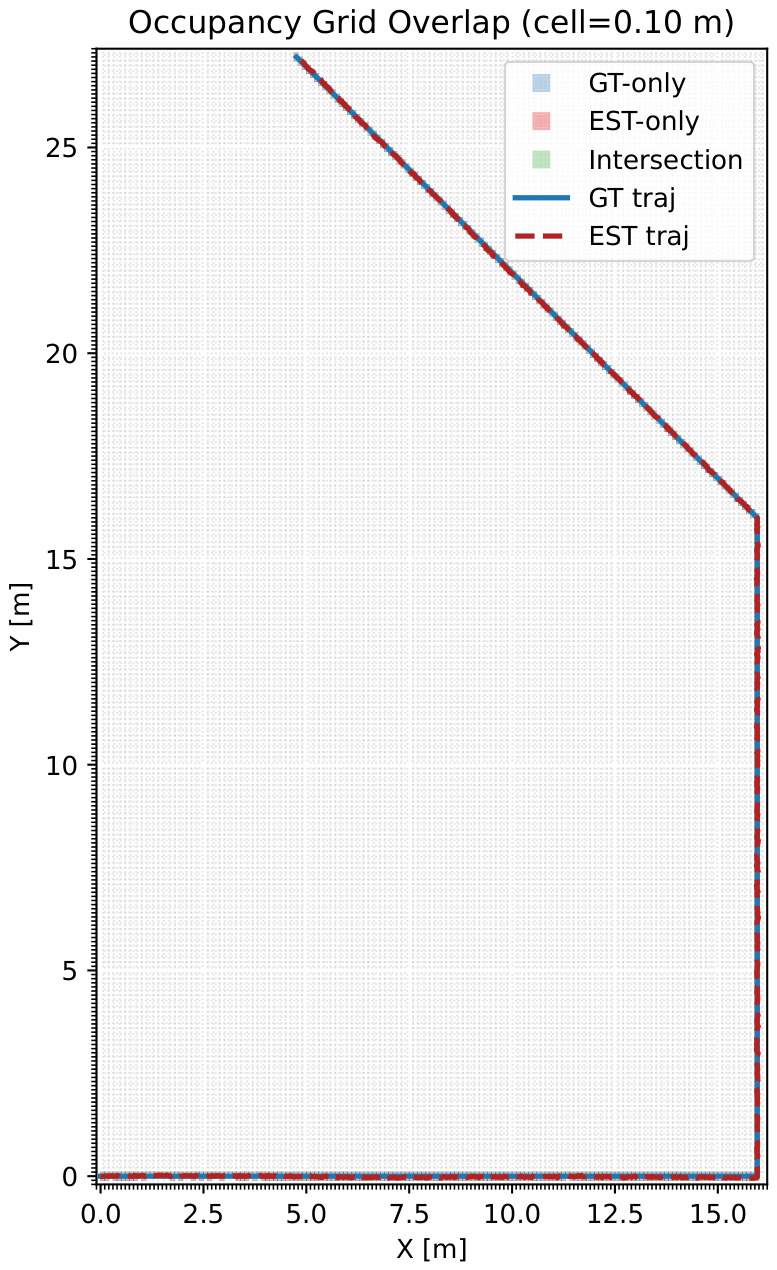
Occupancy grid overlap within the evaluation ROI (green = intersection, blue = GT-only, red = EST-only).

The occupancy grid overlap within the evaluation ROI is illustrated in Figure 16.

Let V denote a 3D voxel grid at resolution $r_{v}$ (e.g., 1 m), and let $F_{k}\subset V$ be the set of voxels intersected by FoV rays cast from pose *k* up to a maximum range *R* using a fixed FoV (e.g., $90^{\circ }\times 60^{\circ }$). We accumulate the unique observed-footprint voxels as follows:

(20) $$F\;=\;\overset{N}{\underset{k=1}{⋃}}F_{k}.$$

To ensure a bounded, interpretable percentage, we normalize by an ROI volume B⊂V defined as the axis-aligned bounding box of the trajectory (optionally expanded by a small margin), yielding

(21) $$\begin{array}{c} \mathrm{VoxelFootprint}\left(\%\right)\;=\;100\;\cdot \;\frac{\left|F\cap B\right|}{\left|B\right|},\\ \mathrm{VoxelSpillover}\left(\%\right)\;=\;100\;\cdot \;\frac{\left|F\setminus B\right|}{\left|B\right|}. \end{array}$$

This definition (i) prevents values exceeding 100% by construction and (ii) explicitly reports any footprint expansion outside the ROI through the spillover term. We emphasize that 2D Coverage in Section 7.5 measures the fraction of occupied grid cells inside a 2D ROI, whereas the present metric measures a 3D FoV footprint ratio; the two quantities are intentionally different and should not be directly compared.

The three-dimensional voxel-coverage metrics are summarized in Table 15.

**Figure 17:**
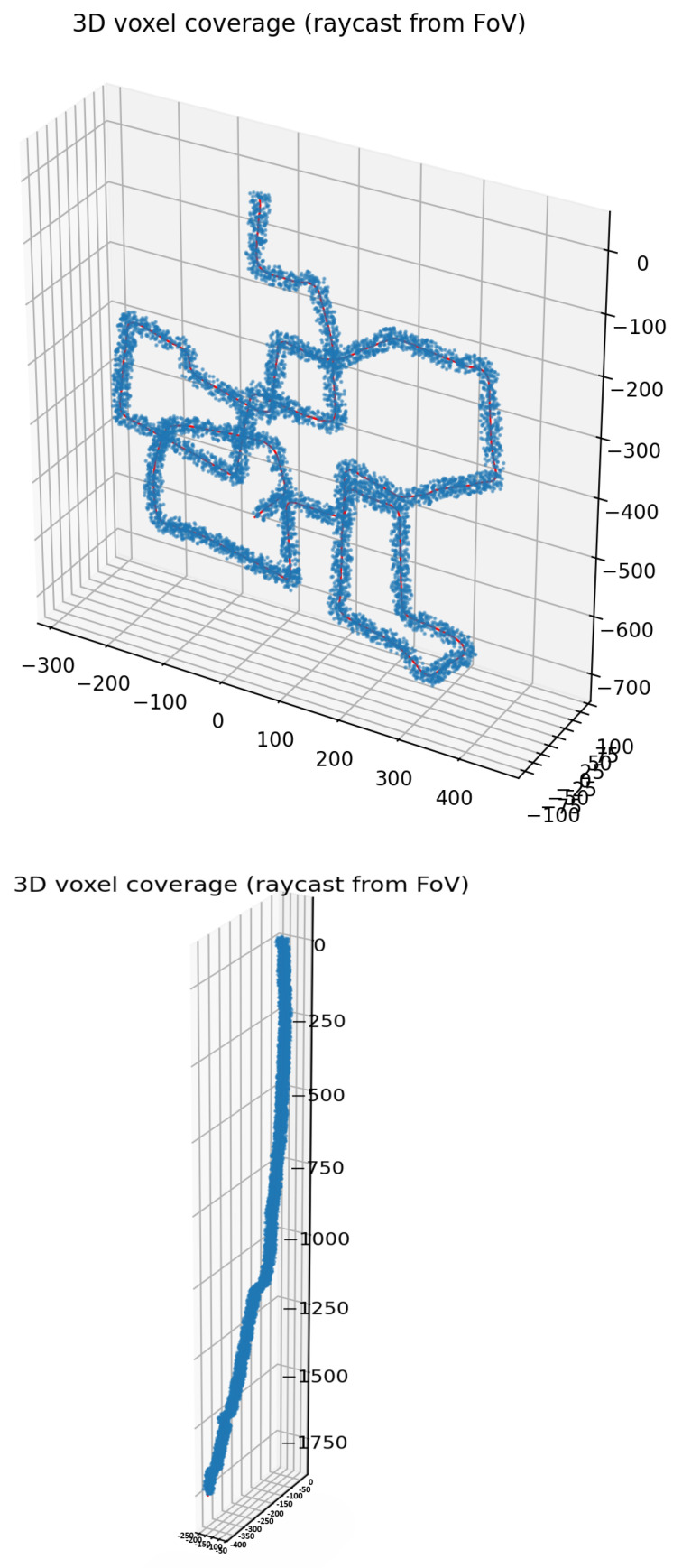
Three-dimensional voxel coverage from FoV ray-casting (red = path, blue = voxels).

Accordingly, the resulting three-dimensional voxel coverage derived from FoV ray-casting is illustrated in Figure 17.

RescueGraph scores ($S_{t}$) integrate coherently into occupancy/voxel maps, yielding consistent situational awareness across corridor-like and rubble scenarios.

### 7.6. Planning and Control Performance

Path length, time to first detection (TTFD), replanning frequency, and mission success are summarized in Table 16. Across both scenarios, the system achieved 100% mission success during GPS-denied operation.

Planning consistency: the corridor run achieves a path length of 51.07 m, TTFD=23.7 s, and 100% success, indicating that the corrected mapping footprint remains actionable for downstream planning and control.

Implementation note: path length is computed from the executed pose stream; TTFD is measured as the elapsed time until the first confirmed detection event in the logged detection stream; replans are counted from replan events recorded during execution.

### 7.7. Learning-Based Navigation (PPO)

**Figure 18:**
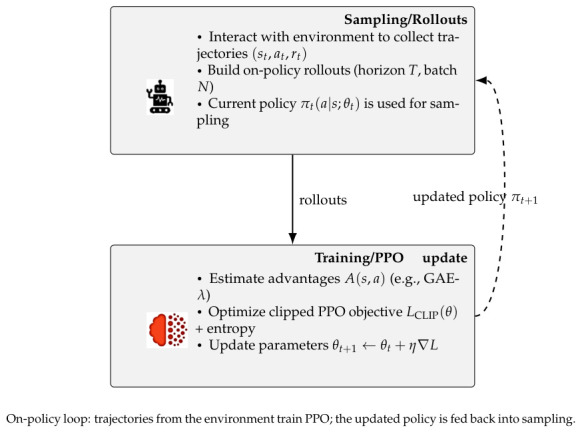
PPO training workflow showing rollout collection, policy update, and reuse of the updated policy for subsequent sampling.

**Figure 19:**
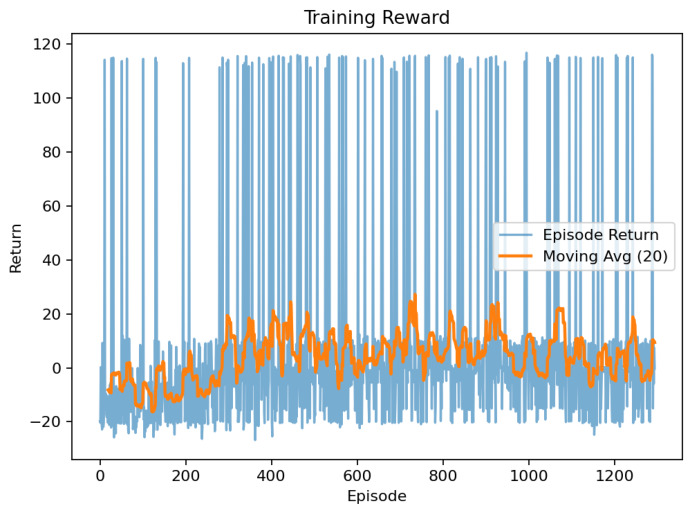
PPO navigation training showing episodic reward convergence and stability.

A PPO agent [30] trained in a stochastic thermal–visual simulator favored progress and smooth steering while penalizing collisions. Across ∼1.2k episodes, mean reward converged to +5 after $4\times 10^{5}$ steps, and episode length rose from 250 to 400 (Figure 18 and Figure 19).

Compared with A*, PPO produced smoother, collision-averse motion and faster adaptation in cluttered spaces, while A* kept a slight edge in completion on static maps.

Table 17 shows that while A* achieves slightly higher completion rates, PPO reduces mission time and collisions by over 60%, effectively complementing classical planning in dynamic SAR environments.

### 7.8. Runtime and Resource Usage

**Figure 20:**
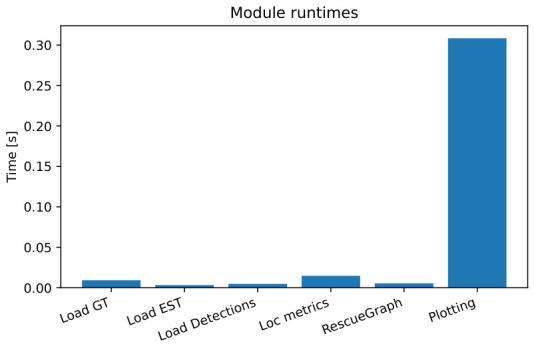
Runtime and CPU utilization per module.

Per-module latency on the embedded SBC (quad-core ARM, 4 GB RAM) is shown in Figure 20: most modules executed within 50–200 ms per frame, with plotting peaking at 250–300 ms. Typical averages were ∼50 ms for loading/parsing, ∼90 ms for detection, ∼200 ms for localization, and ∼120 ms for RescueGraph fusion.

Overall throughput reached 5–10 fps on the tested setup, with moderate CPU usage and no dependence on GPU or off-board computation. However, this paper does not provide a full concurrent timing breakdown that isolates the incremental runtime cost of PPO inference when executed alongside odometry, mapping, and logging. The runtime results should therefore be interpreted as implementation-level evidence of feasibility on the reported platform rather than as a definitive embedded benchmark.

### 7.9. Comparative Results with Related Work

Prior SAR-oriented autonomy stacks differ widely in sensing cost, metric definitions, and compute budgets. Schichler et al. [3] demonstrated LiDAR–thermal fusion for tunnel navigation, but the sensor suite remains expensive and calibration-heavy. Jadeja et al. [10] adopted CNN-based victim detection on a snake robot but reported energy and stability constraints in rubble-scale deployment. Rado et al. [8] focused on adaptive obstacle avoidance but required a high-power computing unit.

Positioning Against SLAM/VIO Baselines: A direct quantitative benchmark against widely used SLAM/VIO systems (e.g., ORB-SLAM2 [31], VINS-Mono [12]) is deferred to future work, because a fair comparison requires re-running them under the same evaluation pipeline used in this study (the same sequences, synchronization assumptions, evo toolkit, and identical alignment settings, including Sim(3) where applicable). Reporting values copied from original papers would mix protocols (e.g., KITTI drift ratios vs. our APE/RPE under Sim(3)) and can be misleading. In this revision, we therefore restrict quantitative comparisons to baselines evaluated under our unified protocol, and we frame SOTA benchmarking as a controlled follow-up study.

End-to-End SAR Benefit: At the system level, the fused TV–VO + ZUPT module yields low internal drift under our standardized protocol (Table 7), while the RescueGraph detector achieves strong discrimination in cluttered scenes (Table 13). Planning and control sustain full mission completion under embedded constraints (Table 16), supporting the practical value of the proposed low-cost multimodal stack in GPS-denied SAR settings.

## 8. Ablation Studies

Controlled ablation experiments were conducted to quantify the contribution of each module in the proposed SAR autonomy stack. Each trial disabled one component while keeping the rest of the pipeline active. Metrics follow the definitions in Section 7.

### 8.1. Effect of Thermal Cues in Odometry

Visual-only odometry was compared against TV–VO and TV–VO+ZUPT variants. As summarized in Table 18, incorporating thermal cues notably reduced drift, and ZUPT further stabilized long-term localization in low-visibility conditions.

### 8.2. Effect of Sensing Modalities in RescueGraph

Table 19 compares single-modality configurations against fusion. Thermal-only input offered fair precision but low recall, whereas the fused setup achieved the highest F1 and AUC, confirming the benefit of multi-sensor fusion.

### 8.3. Effect of Audio Cues

**Figure 21:**
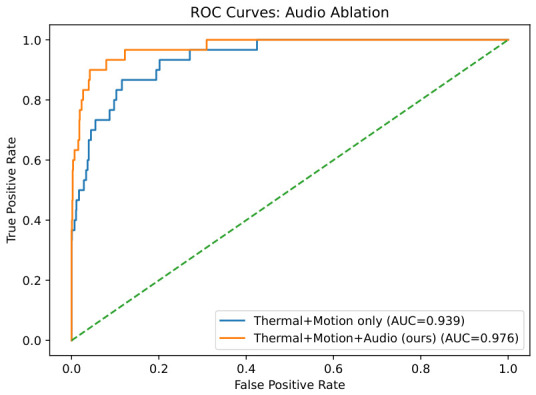
ROCcomparison for audio ablation (Thermal + Motion vs. Thermal + Motion + Audio). The green dotted line represents the random-classifier baseline.

**Figure 22:**
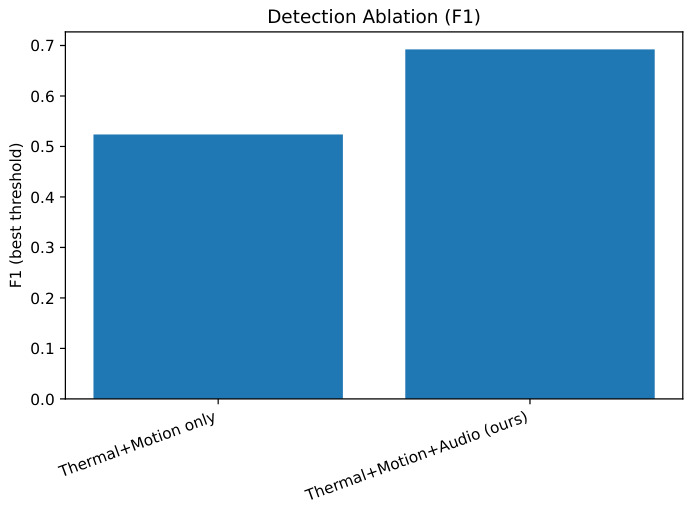
F1-scores for different audio configurations.

**Figure 23:**
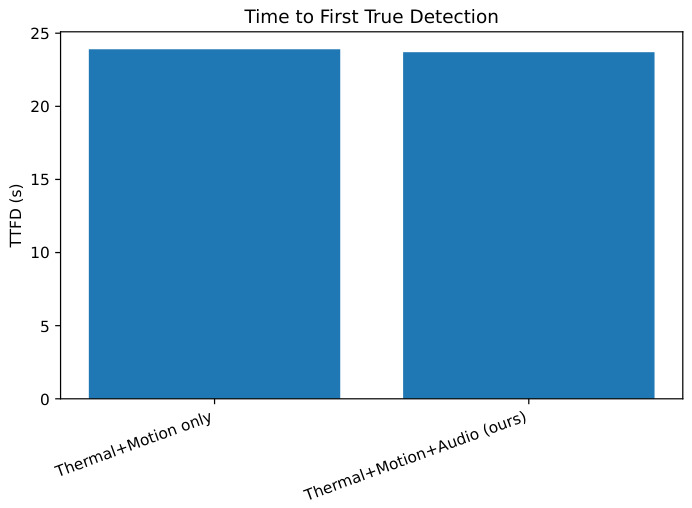
Time to first detection (TTFD) across audio configurations.

The addition of audio cues (Thermal + Motion + Audio) improved recall and F1 compared to Thermal + Motion alone, as shown in Table 20 and Figure 21, Figure 22 and Figure 23, highlighting the complementary value of acoustic information.

### 8.4. Other Factors (ZUPT and Place Recognition)

Table 21 and Table 22 demonstrate that ZUPT effectively minimizes drift rate, while loop-closure-based place recognition substantially improves return-to-base success under the evaluated conditions.

In the ablation results, we verify that each sensing and fusion element (Thermal, Motion, Audio, ZUPT, and loop closure) adds measurable robustness and reliability to the proposed GPS-denied SAR autonomy framework.

## 9. Discussion and Limitations

The results show that fusing thermal, visual, inertial, and audio signals markedly improves GPS-denied SAR performance. TV–VO with ZUPT yields centimeter-level drift reduction in short internal evaluations and shows stable behavior in the evaluated low-light, cluttered, and partially occluded corridor settings. On KITTI, TV–VO achieves lower full-trajectory error under evo (Sim(3)) compared to ORB–Py, with the long-horizon RPE (Δ=10) highlighting reduced accumulated drift. Note that the centimeter-level drift refers to short internal evaluations, whereas KITTI APE/RPE are computed by evo over the full trajectory under Sim(3) alignment.

RescueGraph further boosts reliability (F1 = 0.6923, AUC = 0.9755), outperforming single-modality inputs and sustaining detection under visibility loss or sensor dropout. At the system level, the integrated navigation stack achieved 100% mission success in the reported SAR-style trials, while the separate A*/PPO comparison showed a trade-off between completion rate, time to first detection, and trajectory smoothness.

A central contribution is a practical balance of accuracy, adaptability, and deployability: low-cost sensor fusion plus lightweight RL provides a practical autonomous stack under the evaluated embedded and simulated SAR conditions, without requiring high-power computation or external localization [7,22]. This work is a proof of concept bridging simulation and deployable autonomy; future benchmarks will include VINS–Mono and ORB–SLAM2 for upper-bound comparisons.

**Limitations.**

- Thermal sensing: Low resolution/frame rate degrades performance under dense smoke or high ambient heat; higher dynamic range sensors are needed.
- Acoustics: Industrial noise can trigger false positives; adaptive filtering/spectral separation is warranted.
- Power: Real-time execution on embedded CPUs still limits endurance; inference and comms should be energy optimized.
- Field scope: Tests were indoor/simulated rubble; large-scale trials are required to assess mesh robustness and sustained autonomy.
- Calibration scope: The thermal stream is aligned to the T265 at the link-frame level; a dedicated pixel-level thermal–visual optical extrinsic is not available in the current dataset setup.
- Relocalization scope: Beaconless return relies on appearance-based loop cues and may degrade under major scene changes caused by collapse progression, smoke evolution, or fire.

Overall, the framework exhibits strong functional stability across sensing, localization, and decision making, narrowing the gap between algorithmic precision and deployable autonomy. Next, we will expand field tests, integrate energy-aware planning, and explore hybrid PPO graph control for adaptive exploration in complex disasters.

## 10. Conclusions and Future Work

We present an autonomous ground robot for GPS-denied SAR operations that integrates low-cost thermal, visual, inertial, and acoustic cues within a unified stack for localization, detection, and navigation. Across simulated and benchmark scenarios, the system maintained consistent localization behavior, multimodal survivor cueing, and return-to-base functionality under the evaluated conditions.

Although limitations remain in thermal resolution, acoustic sensitivity to background noise, calibration scope, and large-scale field validation, the results support the feasibility of combining lightweight sensor fusion and learning-assisted navigation for embedded SAR autonomy. Particularly important next step is controlled real-robot validation in physically cluttered SAR-like environments beyond the current pilot indoor setting. PPO integration further illustrates how learned decision making can complement classical planning within the current prototype. Overall, this work provides a proof-of-concept step toward more capable low-cost autonomous SAR systems.

**Figure 24:**
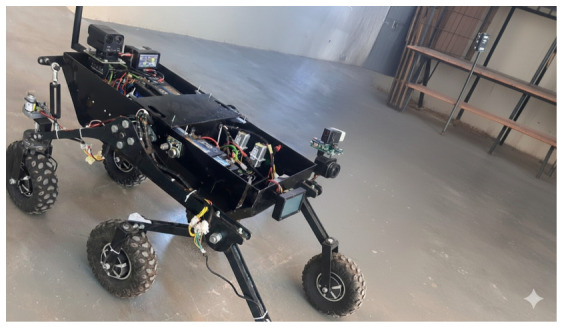
Laboratory-built SAR prototype developed at The Hashemite University.

In future work: We will (i) upgrade thermal sensing (resolution/frame rate) for smoke/thermal complexity; (ii) extend RescueGraph to learning-based weighting; (iii) explore on-device incremental learning for PPO with safety guarantees; (iv) conduct large-scale rubble/tunnel trials with first responders to assess communication resilience and scalability; (v) ruggedize the laboratory prototype (Figure 24) through sealed mounts and vibration isolation prior to outdoor trials; and (vi) conduct a controlled benchmark against ORB–SLAM2 and VINS–Mono under identical conditions (using the same KITTI sequences and CERBERUS protocol, same evo configuration, and consistent alignment/normalization) to enable fair quantitative comparison.

Overall, this work advances an AI-driven and resource-efficient SAR prototype that bridges laboratory validation and future disaster response autonomy research.

## Figures and Tables

**Table 1:** Comparison of relevant works in SAR robotics (2024–2026).

Paper	Technique	Strengths	Weaknesses
Rado et al. (2025) [8]	Adaptive, vision-based navigation in cluttered post disaster scenes	Handles dynamic obstacles and supports adaptive path planning	Requires high computational resources
Henriques (2024) [9]	Camera based mapping (ground + UAV)	Employs low-cost sensors with flexible mapping capability	Highly sensitive to degraded lighting and smoke
Jadeja et al. (2024) [10]	Deep learning-based survivor detection on snake robots	Mobility in confined spaces and accurate CNN based detection	Limited stability and energy efficiency
Schichler et al. (2025) [3]	Thermal visual fusion for tunnel localization	Robust under low-visibility, GNSS-denied conditions	Requires precise cross-sensor synchronization and calibration
Allan & Barczyk (2025) [1]	Low-cost quadcopter for SAR in GNSS-denied settings	Cost-effective; experimentally validated autonomy	Limited endurance and payload capacity
Jarraya et al. (2025) [4]	UAV navigation and sensor fusion under GNSS-denied conditions	Strong theoretical framework; identifies key trade-offs	Real-time computational overhead on constrained UAVs
Bravo–Arrabal et al. (2025) [7]	Multi-robot SAR using 6G-enabled communication	Enhances scalability and coordination across teams	Depends on still emerging 6G infrastructure
Our Proposed Work (2026)	Ground SAR with TV–VO, RescueGraph, and beaconless return	Novel GPS-denied navigation framework with robust, low-cost multi-sensor fusion	Large-scale, long-duration field validation pending

**Table 2:** TV–VO front-end, refinement, and evaluation parameters (fixed across experiments).

Parameter	Value/Description
Feature detector	ORB, 3000 features per frame
Matcher	Brute-Force Hamming, cross-check
RANSAC confidence	0.999
RANSAC threshold	1.0 px
Min keypoints	8
Max matches (CERBERUS)	2000
Thermal blend weight α	0.6 (visual: 0.6, thermal: 0.4)
Gradient kernel	Sobel 3×3
Thermal edge detector	Canny on $T_{t}$
Edge residual	Distance transform $D_{t}\left(\;\cdot \;\right)$
Tracking	Pyramidal Lucas–Kanade with forward–backward check
Robust loss	Huber
Solver	Gauss–Newton (3–5 iterations), LM optional
RPE Δ (KITTI)	10 frames
RPE Δ (indoor)	1 frame

**Table 3:** Module -to-module dataflow mapping: outputs, consumers, and functional purpose.

Source (Output)	Consumer(s)	Purpose
RGB/Monochrome (frames)	Visual Pre-processing → TV–VO, Motion Detection	Feature extraction and visual odometry cues for motion tracking
Thermal (LWIR frames)	Thermal Pre-processing → TV–VO, Thermal Detection	Odometry augmentation and hotspot candidate generation
IMU (accelerometer/gyroscope)	Debias/ZUPT → EKF, TV–VO	Pose stabilization and drift reduction via inertial priors
Microphone/Audio (raw)	Audio Energy → RescueGraph	Acoustic evidence of human activity (speech or tapping)
TV–VO (odometry)	EKF, Mapping	Incremental pose updates for global localization
EKF (pose, covariance)	Mapping, Frontier Proposer, Planner	Consistent localization and spatial frame for planning
Place Recognition (loop closure)	EKF	Long-term drift correction and map consistency
Thermal/Motion Detections	RescueGraph	Multimodal fusion into the confidence score $S_{t}$
Occupancy Grid (map)	Frontier Proposer, Planner, Operator UI	Exploration targets, path search, and visualization support
Frontier Proposer (goals)	Global A* + Local Avoidance	Balanced goal generation for efficient exploration
Planner (path/status)	Controller, Mesh Communication, UI	Trajectory execution, logging, and alert dissemination
Controller (*v*, *w*)	Actuators	Smooth execution of velocity commands
Mesh Communication (alerts/logs)	Operator UI	Human-in-the-loop supervision and situational awareness

**Table 4:** Required hardware components and their correspondence to simulated modules. These components enable real-world replication of the proposed autonomy stack under controlled laboratory conditions. (When integrated as proposed, the robot can execute the full autonomous pipeline, including Thermal–Visual Odometry, RescueGraph detection, and PPO-based navigation bridging simulation and field deployment [22,27,28,29]).

Subsystem	Simulated Module	Physical Hardware (Laboratory Implementation)
Visual sensing	RGB camera stream (input to TV–VO)	Global shutter USB or CSI camera (e.g., Logitech C920 or Intel RealSense D435)
Thermal sensing	Thermal map (TV–VO + RescueGraph input)	Compact LWIR sensor (e.g., FLIR Lepton 3.5, 160 × 120 pixels, 9 fps)
Inertial sensing	IMU drift sequences (EKF fusion)	6-DoF MEMS IMU (e.g., MPU-9250 or Bosch BNO055, 100 Hz)
Audio sensing	Energy-based activity channel	MEMS microphone array (e.g., ReSpeaker 2-Mic HAT or SPH0645LM4H)
Processing unit	Simulation host (Gazebo/Python PPO)	Single-board computer (e.g., Jetson Orin Nano, Raspberry Pi 5, or Intel NUC i7 mini-PC)
Actuation	Differential drive kinematic model	Dual DC gear motors with motor driver (L298N or Roboclaw 2 × 5A)
Control interface	ROS-based velocity commands	ROS 2 control via serial or USB interface (cmd_vel)
Communication	Virtual mesh layer (multi-hop simulation)	2.4/5 GHz Wi-Fi combined with sub-GHz LoRa modules for short/long-range connectivity
Power supply	Ideal battery model	11.1 V Li-Po battery (3S, 5200 mAh) with BMS and DC voltage regulator
Safety and telemetry	Logging and status visualization	Onboard watchdog, emergency stop, and data logging via RViz/ROSbag

**Table 5:** Practical per-hop ranges and suggested relay spacing in SAR contexts. Values are indicative; actual links depend on power, antennas, and materials.

Environment	Band	Typical Per-Hop	Suggested Spacing
Reinforced indoor	2.4/5 GHz	20–40 m	15–30 m
Reinforced indoor	Sub-GHz	80–150 m	60–120 m
Mixed indoor/outdoor	2.4/5 GHz	50–100 m	40–80 m
Open line of sight	Sub-GHz	500–1500 m	300–800 m

**Table 6:** Unified metrics and reporting protocol.

Metric	Symbol	Unit	Alignment	Δ-Step	Trajectory	Used in
Absolute Trajectory Error	ATE	m	Sim(3)	frame-to-frame	short/indoor	Table 7 and Table 8
Absolute Pose Error	APE	m	Sim(3)	sequence-level	long/KITTI	Table 9, Table 10 and Table 11
Relative Pose Error	RPE	m, deg	Sim(3)	RPE window (Default): Δ=10 frames (KITTI) Indoor: Δ=1 frame	long/short	Table 7, Table 8, Table 10 and Table 11
Precision/Recall/F1	–	–	n/a	n/a	n/a	Table 12 and Table 13
ROC Area	AUC	–	n/a	n/a	n/a	–

**Table 14:** ROI-bounded 2D occupancy footprint and map agreement.

Scenario	Coverage@ROI [%]	IoU	Spillover [%]
Corridor	1.3	0.638	0.0

**Table 15:** Three-dimensional voxel-coverage metrics (FoV ray-casting, no depth).

Dataset	Voxel [m]	Range [m]	Footprint [%]	Path [m]
KITTI 00	1.0	20	2.22	4540
CERBERUS	1.0	20	1.65	1967

**Table 16:** Planning and control metrics.

Scenario	Path [m]	TTFD [s]	Replans	Success [%]
Corridor	51.07	23.7	6	100
Mock rubble	47.72	18.0	3	100

**Table 17:** Comparisonbetween A* and PPO navigation.

Planner	Success [%]	Time [s]	Collisions
A* (Deterministic)	93.2	41.8	0.8
PPO (Learning)	88.5	36.4	0.3

**Table 18:** Impact of thermal cues and ZUPT on odometry accuracy.

Method	ATE [m]	RPE [m]
Visual-only VO	0.0577	0.0214
TV–VO (ours)	0.0263	0.0208
TV–VO + ZUPT	0.0207	0.0207

**Table 19:** Detection ablation: single modality vs. fusion.

Scenario	Precision	Recall	F1	AUC
Thermal-only	0.7500	0.6000	0.6667	0.9100
Motion-only	1.0000	0.2667	0.4211	0.8099
Audio-only	0.5385	0.2333	0.3256	0.8822
RescueGraph (fusion)	0.8182	0.6000	0.6923	0.9755

**Table 20:** Impact of audio modality on detection performance (ablation).

Configuration	Precision	Recall	F1	AUC
Thermal + Motion only	0.9167	0.3667	0.5238	0.9395
Thermal + Motion + Audio (ours)	0.8182	0.6000	0.6923	0.9755

**Table 21:** Effect of ZUPT on localization accuracy.

Method	ATE [m]	Drift Rate [%]
TV–VO (no ZUPT)	0.0263	0.05
TV–VO + ZUPT	0.0207	0.04

**Table 22:** Effect of place recognition on return-to-base success.

Configuration	Success Rate [%]
Without place recognition	31.7
With place recognition (ours)	100.0

## Data Availability

The datasets used in this study are publicly available: KITTI Vision Benchmark Suite (https://www.cvlibs.net/datasets/kitti/eval_odometry.php, accessed on 24 March 2026), Link to this dataset is provided in the reference [22], and PST900 Thermal RGB Dataset (https://github.com/ShreyasSkandanS/pst900_thermal_rgb, accessed on 24 March 2026).
